# Occupational exposure to benzene and mortality risk of lymphohaematopoietic cancers in the Swiss National Cohort

**DOI:** 10.5271/sjweh.4164

**Published:** 2024-07-01

**Authors:** Calvin Ge, Adrian Spoerri, Matthias Egger, Nathaniel Rothman, Qing Lan, Anke Huss, Roel Vermeulen

**Affiliations:** 1 Institute for Risk Assessment Sciences, Utrecht University, The Netherlands.; 2 Institute of Social and Preventive Medicine (ISPM), University of Bern, Switzerland.; 3 School of Social and Community Medicine, University of Bristol, UK.; 4 Occupational and Environmental Epidemiology Branch, Division of Cancer Epidemiology and Genetics, National Cancer Institute, NIH, DHHS, Bethesda, MD, USA.; 5 Julius Centre for Public Health Sciences and Primary Care, University Medical Centre, Utrecht, The Netherlands.

**Keywords:** lymphoma, myeloid leukemia

## Abstract

**Objectives:**

Previous studies established a causal relationship between occupational benzene exposure and acute myeloid leukemia (AML). However, mixed results have been reported for associations between benzene exposure and other myeloid and lymphoid malignancies. Our work examined whether occupational benzene exposure is associated with increased mortality from overall lymphohaematopoietic (LH) cancer and major subtypes.

**Methods:**

Mortality records were linked to a Swiss census-based cohort from two national censuses in 1990 and 2000. Cases were defined as having any LH cancers registered in death certificates. We assessed occupational exposure by applying a quantitative benzene job-exposure matrix (BEN-JEM) to census-reported occupations. Exposure was calculated as the products of exposure proportions and levels (P × L). Cox proportional hazards models were used to calculate LH cancer death hazard ratios (HR) and 95% confidence intervals (CI) associated with benzene exposure, continuously and in ordinal categories.

**Results:**

Our study included approximately 2.97 million persons and 13 415 LH cancer cases, including 3055 cases with benzene exposure. We observed increased mortality risks per unit (P x L) increase in continuous benzene exposure for AML (HR 1.03, 95% CI 1.00–1.06) and diffuse large B-cell lymphoma (HR 1.09, 95% CI 1.04–1.14). When exposure was assessed categorically, increasing trends in risks were observed with increasing benzene exposure for AML (P=0.04), diffuse large B-cell lymphoma (P=0.02), and follicular lymphoma (P=0.05).

**Conclusion:**

In a national cohort from Switzerland, we found that occupational exposure to benzene is associated with elevated mortality risks for AML, diffuse large B-cell lymphoma, and possibly follicular lymphoma.

Benzene is a ubiquitous air pollutant. In the workplace, benzene exposure occurs in industries such as oil and gas extraction, refinement of petroleum products, and shoe production (1). In Europe and Canada, studies have estimated that approximately 1–2% of the total working population was exposed to benzene in the workplace (2, 3).

The International Agency for Research on Cancer (IARC) classifies benzene as a group 1 carcinogen in humans (1, 4). Association between occupational benzene exposure and acute myeloid leukemia (AML) was observed in a number of studies, including recent industrial cohort studies with quantitative exposure estimates (5–7). However, the relationship between benzene exposure and other lymphohaematopoietic (LH) cancers is less clear (1, 4).

For the present study, we assessed historical occupational benzene exposure for more than 2.97 million persons in the Swiss National Cohort using a quantitative benzene job-exposure matrix (JEM). We evaluated the associated mortality risks from overall LH cancer and major subtypes, including AML, chronic myeloid leukemia (CML), non-Hodgkin lymphoma (NHL), acute lymphoid leukemia (ALL), chronic lymphoid leukemia (CLL), diffuse large B-cell lymphoma (DLBCL), small cell B-cell lymphoma (SCBCL), mantle cell lymphoma (MCL), T/NK-cell lymphoma (T/NK-NHL), and follicular lymphoma (FL), multiple myeloma (MM), and Hodgkin lymphoma (HL).

## Methods

### Study population

The Swiss National Cohort is a longitudinal study based on two national censuses in Switzerland in 1990 and 2000. Records from the censuses were linked with mortality and emigration records using deterministic and probabilistic methods (8, 9). Key variables used in the linkages include sex, date of birth, marital status, nationality, religion, place of residence, spousal information, and family structure. We excluded persons <30 years from our analysis because census linkage for these subjects was less complete due to their higher mobility and likelihood of living alone (8) and the low mortality rate among these subjects.

Standardized registration of deaths and participation in the national census were mandatory in Switzerland. For instance, for the year 2000, the census coverage was 98.6% (10), and 94% of all deaths could be linked to census records. The Swiss National Cohort study obtained ethics approval from the Cantonal Ethics Committees of Bern. Additional information on the cohort and study is available online at www.swissnationalcohort.ch.

### Exposure assessment and case definition

Census participants, by self-enumeration, reported approximately 18 000 distinct jobs in the two Swiss censuses. The Swiss National Cohort dataset already included standardized occupation coding using the 1988 version of the International Standard Classification of Occupations (ISCO-88), assigned by the Swiss Federal Office and reviewed by one of the authors (AH). Occupations reported by census participants when they entered the risk period (either in 1990 or 2000) were used for exposure assessment. We estimated occupational exposure to benzene using the BEN-JEM, a quantitative general population JEM developed by one of the authors (RV) (11, 12). For each ISCO-88 occupation, the BEN-JEM assesses the proportion of workers exposed (P) and the mean level of benzene exposure (L) in parts per million (ppm). The BEN-JEM also assigns different proportions and levels of exposure for different time periods, taking into account the decreasing trend of occupational benzene exposure in Europe and North America over time (13). BEN-JEM has estimates for the periods 1945–1959; 1960–1984; 1985–1994; 1995–1997; 1998–2000; 2001–2003; 2004–2006; and 2007–2009; relevant BEN–JEM assessment periods for our study include 1985–1994 and 1998–2000. For instance, a subject with an occupation in both 1990 and 2000 would be assigned the respective exposure levels at the corresponding point in time. Exposure for a particular job was calculated as the products of exposure proportions and levels (P × L). We also assessed exposure with ordinal exposure categories. Benzene exposures were categorized as low, medium, and high when the P × L was <2, 2–10, and >10, respectively (11). Jobs with zero exposure proportion or level were categorized as unexposed.

Cases were defined as deaths from LH neoplasms registered anywhere on death certificates. Our choice to use LH neoplasms as registered anywhere on death certificates was intended to maximize the capture of rare outcomes, as this is particularly key in obtaining sufficient cases for analysis of some of the very rare LH neoplasms, such as FL and MCL. Versions 8 and 10 of the International Classification of Diseases and Related Health Problems (ICD8/10) were used to identify LH cancer categories and subtypes. Causes of death were recorded in Switzerland using ICD8 before 1995 and ICD10 since 1995. Disease categories and associated ICD codes used in our study are listed in table 1.

**Table 1:** Lymphohaematopoietic cancer and subtypes of interest with corresponding ICD8/10 codes, as registered anywhere on death certificate [ICD8/10=International Classification of Diseases and Related Health Problem version 8/10; AML=acute myeloid leukaemia; CML=chronic myeloid leukaemia; ALL=acute lymphoid leukaemia; CLL=chronic lymphoid leukaemia; FL=follicular lymphoma; DLBCL=diffuse large B-cell lymphoma; SCBCL=small cell B-cell lymphoma; MZL=marginal zone lymphoma; LPL=lymphoplasmacytic lymphoma; WM=Waldenström’s macroglobulinemia]

Disease category				ICD8	ICD10
Lymphohaematopoietic cancer				200-209	C81-C96
	Myeloid leukaemia			205	C92
		AML		205.0	C92.0
		CML		205.1	C92.1
	Lymphoid neoplasms			200-204	C81-C91
		Non-Hodgkin Lymphoma		200, 202, 204	C82-C91
			ALL	204.0	C91.0
			CLL	204.1	C91.1
			DLBCL	-	C83.3
			SCBCL (including MZL, LPL, & WM)	-	C83.0 + C88.0
			MCL		C83.1
			T/NK-NHL		C84 + C86
			FL	202.0	C82
		Multiple myeloma		203	C90
		Hodgkin lymphoma		201	C81

### Statistical analysis

We used Cox proportional hazard models to evaluate the associations between occupational benzene exposure and LH cancer-related deaths. Our models used participant age as the underlying time scale. For the main analysis, participants entered the risk set on 4 December 1990, the day of the 1990 census. Persons <30 years or who immigrated to Switzerland between 4 December 1990 and 4 December 2000 entered the risk set on 4 December 2000, the day of the 2000 census. Observation time ended on the earliest of the following: date of death, date of emigration, or 31 December 2016. For disease categories introduced in ICD10 (ie, DLBCL, SCBCL, T/NK-NHL, and MCL), subjects entered the risk period on 1 January 1995, when ICD10 use began in Switzerland. We adjusted for sex in the minimally adjusted model and additionally adjusted for nationality, education, language region, and marital status in the full model. We stratified analyses to account for differences in baseline mortality risk over time (“strata” command in Stata, stratified by the census 1990 and 2000).

For models with benzene exposure as a categorical variable, we assessed trends in the risk estimates by calculating the P-value in a likelihood ratio test after setting exposure categories to values 0, 1, 2, and 3. If the number of cases in the high exposure category was less than five, we combined the medium and high exposure categories into one “medium/high” exposure category. No risk estimates were calculated for health outcomes with less than five cases in any exposure categories. We tested models for proportionality assumption using statistical tests based on Schoenfeld residuals, and the assumption was met for all exposure variables. All analyses used Stata version 14 (StataCorp, College Station, TX, USA).

## Results

The 1990 census included 3055 million persons aged between 30 and retirement age (62 and 65 years for women and men, respectively). We excluded 577 226 persons with missing or uncertain occupational information (ie, ISCO-88 coded to major group only), 64 766 pensioners, and 255 251 people who were homemakers or unemployed, leaving 2158 million persons for the analysis restricted to 1990–2000. The 2000 census had 3470 million persons aged between 30 and retirement age. We excluded 836 270 persons with missing or uncertain occupational information, 100 947 persons on pension, and 498 414 people who were homemakers or unemployed, leaving 2035 million persons for the analysis restricted to 2000–2016. For our final combined 1990–2016 analyses, 2976 million persons were included.

Across our study population, 19 926 deaths from LH cancers occurred in the risk period, of which 98% could be linked to a census record. Of the 19 539 census-linked cases, we excluded 3714 cases because they had uncertain occupational information, 1106 cases because they were pensioners, and 1304 cases because they were homemakers or unemployed. A total of 13 415 LH cases were included in our final analysis, including 3055 cases with assessed occupational benzene exposure. Characteristics of our study population at baseline are shown in table 2.

**Table 2:** Study population characteristics at baseline (4 December 1990).

		Occupational benzene exposure level ^a^						
		None (N=1 655 412)		Low (N=234 586)		Medium (N=246 741)		High (N=20 775)
		%		%		%		%
Female		43		34		29		2
Education level ^b^								
	Low	18		36		23		19
	Medium	53		45		63		66
	High	29		19		14		15
Foreign nationals		17		36		29		40
Language region								
	German	72		74		74		73
	French	24		23		22		23
	Italian	4		3		4		4
Marital status								
	Single	12		11		11		12
	Married	72		76		76		75
	Widowed	4		4		3		2
	Divorced	12		10		10		11

^a^ Exposure=sum of the product of the proportions of workers exposed and level of exposure in ppm (P × L) across all occupations reported at censuses. Low, medium, and high exposure groups correspond to P × L values of <2, 2–10, and >10, respectively. ^b^ Low=compulsory education or less; medium=upper secondary level education; high=tertiary level education.

Increased hazard ratios (HR) for AML were observed in both the minimally and fully adjusted continuous benzene exposure model [HR 1.03 per P × L unit exposure increase, 95% confidence interval (CI) 1.00–1.06 in both models] (table 3). HR for DLBCL also showed an association with continuous benzene exposure (HR 1.09 per P × L unit exposure increase, 95% CI 1.04–1.14 in both models).

**Table 3:** Hazard ratios (HR) and confidence interval (CI) for mortality from LH cancers and subtypes with benzene exposure ^a^ as a continuous variable for persons in the Swiss National Cohort [LH=lymphohaematopoietic; AML=acute myeloid leukaemia; CML=chronic myeloid leukaemia; NHL=non-Hodgkin lymphoma; ALL=acute lymphoid leukaemia; CLL=chronic lymphoid leukaemia; DLBCL=diffuse large B-cell lymphoma; SCBCL=small cell B-cell lymphoma; WM=Waldenström’s macroglobulinemia; FL=follicular lymphoma; MCL=mantle cell leukaemia; T/NK-NHL=mature T/NK cell lymphoma; MM=multiple myeloma; and HL=Hodgkin lymphoma]

				Minimally adjusted ^b^ HR (95% CI)	Fully adjusted ^b^ HR (95% CI)
Any LH cancer				1.00 (0.99–1.02)	1.00 (0.99–1.01)
	Myeloid leukaemia			1.02 (1.00–1.04)	1.01 (0.99–1.04)
		AML		1.03 (1.00–1.06)	1.03 (1.00–1.06)
		CML		1.02 (0.96–1.08)	1.01 (0.95–1.08)
	Lymphoid neoplasms			1.00 (0.98–1.01)	1.00 (0.98–1.01)
		NHL		1.00 (0.98–1.02)	1.00 (0.98–1.02)
			ALL	0.96 (0.84–1.90)	0.96 (0.84–1. 09)
			CLL	0.99 (0.95–1.03)	0.99 (0.95–1.03)
			DLBCL	1.09 (1.04–1.14)	1.09 (1.04–1.14)
			SCBCL (including WM)	0. 90 (0.79–1.03)	0.89 (0.77–1.02)
			MCL	0.83 (0.65–1.06)	0.84 (0.66–1.06)
			T/NK-NHL	0.98 (0.91–1.06)	0.98 (0.91–1.06)
			FL	1.06 (0.98–1.14)	1.05 (0.97–1.14)
	MM			0.99 (0.96–1.02)	0.99 (0.96–1.02)
	HL			1.04 (0.98–1.10)	1.04 (0.97–1.10)

^a^ Exposure=sum of the product of the proportions of workers exposed and level of exposure in ppm (P × L) across all occupations reported at censuses. ^b^ Minimally adjusted model was adjusted for sex, and fully adjusted model additionally adjusted for nationality, education, language region, and marital status

Based on the fully adjusted models with categorical benzene exposure, increasing risk trends for AML (P=0.04), DLBCL (P=0.02), and FL (P=0.05) were observed with increasing benzene exposure (table 4). Elevated risk point estimates were observed for AML for the medium (HR 1.11, 95% CI 0.97–1.27) and high exposure group (HR 1.35, 95% CI 0.90–2.03); however, these increases were associated with larger uncertainties. HR for DLBCL was 1.22 (95% CI 0.94–1.59) for the medium exposure group and 2.78 (95% CI 1.52–5.09) for the high exposure group. We also observed increased risks for FL for the combined medium/high exposure group (HR 1.58, 95% CI 1.09–2.29) and HL for the low exposure group (HR 1.37, 95% CI 1.03–1.82).

Elevated HR point estimates were observed for some other LH cancers in our fully adjusted models. HR for myeloid leukemia (AML + CML) was 1.09 (95% CI 0.96–1.22) for the medium exposure group and 1.06 (95% CI 0.72–1.57) for the high exposure group. For CML, HR was 1.06 (95% CI 0.81–1.39) for the medium exposure group and 1.08 (95% CI 0.44–2.61) for the high exposure group.

**Table 4:** Hazard ratios (HR) and 95% confidence intervals (CI) for mortality from LH cancers and subtypes with benzene exposure a as a categorical variable for persons in the Swiss National Cohort .[LH=lymphohaematopoietic; AML=acute myeloid leukaemia; CML=chronic myeloid leukaemia; NHL=non-Hodgkin lymphoma; ALL=acute lymphoid leukaemia; CLL=chronic lymphoid leukaemia; DLBCL=diffuse large B-cell lymphoma; SCBCL=small cell B-cell lymphoma; WM=Waldenström’s macroglobulinemia; FL=follicular lymphoma; MCL=mantle cell leukaemia; T/NK-NHL=mature T/NK cell lymphoma; MM=multiple myeloma; and HL=Hodgkin lymphoma].

		N	Minimally adjusted ^b^ HR (95% CI)	Fully adjusted ^b^ HR (95% CI)	P-trend
Any LH cancer					0.32
	Unexposed	10 360	referent	referent	
	Ever exposure	3055	1.01 (0.98-1.05)	1.00 (0.97-1.04)	
	Low exposure	1333	1.00 (0.95-1.06)	1.00 (0.94-1.06)	
	Medium exposure	1602	1.03 (0.98-1.09)	1.01 (0.96-1.07)	
	High exposure	120	1.00 (0.84-1.20)	0.99 (0.83-1.19)	
Myeloid leukemias					0.11
	Unexposed	2102	referent	referent	
	Ever exposure	636	1.07 (0.98-1.16)	1.06 (0.97-1.16)	
	Low exposure	273	1.03 (0.91-1.17)	1.03 (0.90-1.17)	
	Medium exposure	337	1.10 (0.98-1.24)	1.09 (0.96-1.22)	
	High exposure	26	1.09 (0.74-1.60)	1.06 (0.72-1.57)	
AML					0.04
	Unexposed	1554	referent	referent	
	Ever exposure	472	1.09 (0.99-1.21)	1.09 (0.99-1.20)	
	Low exposure	200	1.03 (0.89-1.20)	1.04 (0.90-1.21)	
	Medium exposure	248	1.12 (0.98-1.28)	1.11 (0.97-1.27)	
	High exposure	24	1.39 (0.93-2.08)	1.35 (0.90-2.03)	
CML					0.56
	Unexposed	385	referent	referent	
	Ever exposure	121	1.06 (0.87-1.28)	1.04 (0.86-1.26)	
	Low exposure	52	1.03 (0.77-1.38)	1.01 (0.75-1.35)	
	Medium exposure	64	1.08 (0.83-1.41)	1.06 (0.81-1.39)	
	High exposure	5	1.07 (0.44-2.58)	1.08 (0.44-2.61)	
Lymphoid neoplasms					0.94
	Unexposed	7853	referent	referent	
	Ever exposure	2290	1.00 (0.96-1.05)	0.99 (0.95-1.04)	
	Low exposure	1013	1.00 (0.94-1.07)	1.00 (0.94-1.07)	
	Medium exposure	1189	1.01 (0.95-1.07)	0.99 (0.93-1.05)	
	High exposure	88	0.97 (0.78-1.19)	0.96 (0.78-1.18)	
NHL					0.72
	Unexposed	5206	referent	referent	
	Ever exposure	1531	1.01 (0.95-1.06)	1.00 (0.94-1.05)	
	Low exposure	666	0.99 (0.92-1.08)	0.99 (0.91-1.07)	
	Medium exposure	803	1.02 (0.94-1.10)	1.00 (0.92-1.08)	
	High exposure	62	1.02 (0.79-1.31)	1.01 (0.78-1.29)	
ALL					0.88
	Unexposed	172	referent	referent	
	Ever exposure	41	0.94 (0.68-1.31)	0.94 (0.68-1.31)	
	Low exposure	19	0.96 (0.59-1.54)	0.97 (0.60-1.58)	
	Medium/high exposure	22	0.93 (0.59-1.46)	0.92 (0.58-1.45)	
CLL					0.70
	Unexposed	1028	referent	referent	
	Ever exposure	320	1.00 (0.89-1.13)	0.99 (0.88-1.12)	
	Low exposure	127	0.93 (0.77-1.11)	0.92 (0.76-1.11)	
	Medium exposure	181	1.07 (0.91-1.26)	1.06 (0.90-1.25)	
	High exposure	12	0.93 (0.52-1.64)	0.93 (0.53-1.65)	
DLBCL					0.02
	Unexposed	389	referent	referent	
	Ever exposure	125	1.21 (1.01 -1.47)	1.21 (1.01 -1.47)	
	Low exposure	47	0.98 (0.73-1.33)	0.99 (0.73-1.34)	
	Medium exposure	67	1.24 (0.96-1.62)	1.22 (0.94-1.59)	
	High exposure	11	2.77 (1.51-5.06)	2.78 (1.52-5.09)	
SCBCL (including WM)					0.31
	Unexposed	237	referent	referent	
	Ever exposure	67	0.94 (0.72-1.21)	0.90 (0.70-1.17)	
	Low exposure	32	1.02 (0.71-1.48)	1.00 (0.69-1.45)	
	Medium/high exposure	35	0.86 (0.60-1.24)	0.82 (0.57-1.17)	
MCL					0.89
	Unexposed	121	referent	referent	
	Ever exposure	35	0.93 (0.65-1.33)	0.93 (0.65-1.33)	
	Low exposure	13	0.81 (0.45-1.43)	0.79 (0.44-1.41)	
	Medium/high exposure	22	1.01 (0.64-1.59)	1.03 (0.65-1.64)	
FL					0.05
	Unexposed	159	referent	referent	
	Ever exposure	54	1.31 (0.97 -1.76)	1.27 (0.94 -1.72)	
	Low exposure	16	0.83 (0.49-1.38)	0.83 (0.49-1.40)	
	Medium/high exposure	38	1.64 (1.14-2.36)	1.58 (1.09-2.29)	
T/NK-NHL					0.92
	Unexposed	321	referent	referent	
	Ever exposure	97	1.08 (0.87 -1.34)	1.08 (0.87 -1.35)	
	Low exposure	49	1.23 (0.91-1.66)	1.24 (0.91-1.68)	
	Medium/high exposure	48	0.94 (0.69-1.28)	0.94 (0.69-1.29)	
MM					0.48
	Unexposed	2350	referent	referent	
	Ever exposure	658	0.97 (0.90-1.06)	0.96 (0.89-1.04)	
	Low exposure	293	0.97 (0.86-1.10)	0.97 (0.86-1.10)	
	Medium exposure	344	0.99 (0.88-1.11)	0.97 (0.87-1.09)	
	High exposure	21	0.79 (0.51-1.21)	0.79 (0.51-1.22)	
HL					0.36
	Unexposed	328	referent	referent	
	Ever exposure	110	1.20 (0.98 -1.46)	1.19 (0.97 -1.46)	
	Low exposure	57	1.37 (1.03-1.82)	1.37 (1.03-1.82)	
	Medium exposure	46	0.95 (0.70-1.30)	0.94 (0.69-1.29)	
	High exposure	7	1.79 (0.84-3.79)	1.78 (0.84-3.78)	

^a^ Exposure = sum of the product of the proportions of workers exposed and level of exposure in ppm (P × L) across all occupations reported at censuses. Low, medium, and high exposure groups correspond to P × L values of <2, 2-10, and >10, respectively. ^b^ Minimally adjusted model was adjusted for sex, and fully adjusted model additionally adjusted for nationality, education, language region, and marital status.

## Discussion

For myeloid leukemia and its subtypes, we observed the strongest association between benzene exposure and AML. Increases in AML mortality risks were observed when benzene exposure was assessed both continuously and categorically. Our findings are consistent with the current consensus that benzene exposure is causally linked to AML (1, 4). For the other subtype of myeloid leukemia (CML), we found elevated HR point estimates for persons in the medium and high exposure categories. Our results are similar to several studies on benzene-exposed workers in industrial cohorts that reported elevated risk point estimates for CML, but no clear results for an exposure–response relationship (7, 14–16). A pooled analysis of benzene-exposed petroleum workers reported clear increases in CML risks and an exposure–response trend (5).

Among lymphomas, increases in HR for DLBCL were associated with both continuous and categorical benzene exposure metrics based on 125 exposed cases. Our findings are consistent with results from a study with 40 exposed cases among a cohort of solvent-exposed female workers in the US, which reported increases in DLBCL risks with increases in benzene exposure intensity and probability (17). However, a study of similar design in the Norwegian oil industry male workers observed no association between benzene exposure and DLBCL based on 15 exposed cases (6). Two population-based case-control studies also found no risk increases in DLBCL in relation to occupational benzene exposure. One of these studies was performed in a European cohort assembled from the Czech Republic, France, Germany, Ireland, Italy, and Spain, which included 28 exposed cases (18); the other was from China with 9 exposed cases (19).

Our analyses based on categorical benzene exposure also suggest a possible relationship between benzene exposure and FL based on 54 exposed cases. We also observed elevated FL risks in our continuous exposure models, albeit with larger uncertainties. In a hospital-based case–control study in Shanghai, Wong and colleagues (19) reported an odds ratio of 7.00 (95% CI 1.45–33.70) based on seven exposed cases. Increases in FL risk point estimates were also reported by other studies performed in benzene-exposed workers in industrial cohorts (6, 17) and the general population (18, 20).

Our results do not support an association between benzene exposure and overall NHL mortality risk. A key challenge in comparing results between different studies investigating risks of NHL is the heterogeneous classification of the disease category. Under the current 2008 World Health Organization (WHO) classification scheme, NHL includes subtypes such as ALL, CLL, and MM (21). Historically, ALL and CLL were not classified as subtypes of NHL, which may lead to different findings across studies using other classification schemes. For instance, among three studies conducted in the Chinese population, two reported positive associations between benzene exposure and NHL (16, 22) and one reported no association (19). All three studies, however, used different definitions for NHL: Wong and colleagues (19) included all lymphoid leukemia (ALL; CLL) and MM cases; Bassig and colleagues (22)included ALL and CLL but not MM cases; Linet and colleagues (16) excluded all ALL, CLL, and MM cases. Two other studies reported suggestive associations between benzene and combined B-cell NHL (6, 18); however, this disease category excludes T-cell and NK-cell lymphomas and thus may not be directly comparable to our results for NHL. Our findings suggest benzene exposure may be a subtype-specific risk factor for DLBCL and possibly for FL. Our study, along with several other studies which reported differential NHL subtype risks for benzene-exposed workers (6, 17–20), highlights the importance of assessing and reporting risk by disease subtype when possible, as there may be significant etiologic heterogeneity between different NHL subtypes. This is consistent with evidence supporting different known or suspected lifestyle risk factors for different types of NHL (23). In addition, abundant experimental and human evidence shows that benzene is lymphomagenic and can cause a variety of biological perturbations, including CD4+ T cell toxicity and an increase in lymphocyte chromosomal aberrations (4).

For HL, increased mortality risks were limited to the low benzene exposure group and not observed in our continuous exposure models. Earlier studies generally report no association between benzene exposure and HL (20, 24). In a meta-analysis of lymphoma subtypes by Vlaanderen and colleagues (25), no association was observed between occupational benzene exposure and HL.

There are several limitations to our study. Occupations reported in a single time point (either 1990 census occupation for subjects entering the risk period in 1990 or 2000 census occupation for subjects entering in 2000) were the only information available for assessing occupational benzene exposure. Therefore, our study could not account for any changes in occupation before or after the census dates, leading to potential misclassification of exposure. We also acknowledge that a healthy worker effect may have affected our results. We regard the chance for this to be minimal as there are no strong prolonged prodromal effects of lymphohaematopoietic cancers. The overall exposure misclassification is, therefore, unlikely to be differential and would reduce the magnitude of the observed risk estimates. Applying a JEM for exposure assessment results in the same exposure being used for subjects with the same ISCO-88 job title in the same time period, without consideration for individual differences within the job groups. This produces Berkson-type error, which increases the uncertainties, but does not bias the observed risk estimates (26, 27). Our work included analyses on associations between benzene exposure and several LH cancer outcomes; we therefore cannot rule out some findings may be due to chance from multiple comparisons. LH cancer outcomes were captured through death certificates. Compared with other means of capturing disease outcome, such as incidence registrations, our approach may miss LH cancer cases in the Swiss population, particularly for subtypes with lower mortality. Such omission of cases would reduce statistical power in our study, leading to more uncertain, but unbiased, risk estimates for these LH cancer subtypes. Our choice to exclude subjects under 30 may also result in the exclusion of benzene-exposed cases who died before age 30; though this is likely rare given the group’s low mortality. Although a common practice in occupational epidemiology, the aggregation of exposure probability and levels have been shown to produce bias in simulated datasets under specific conditions (28). While industry information for subjects’ occupations may help improve benzene exposure assessment in certain cases, sector information was neither available from our Swiss National Cohort dataset nor the BEN-JEM. Because the aforementioned limitations will likely lead to attenuation and larger uncertainties of our effect estimates, we chose to analyses results both categorically and continuously to allow for comparison of results by disease subtype and to ensure we are able to detect exposure response associations in our study. Overall, the fact that the established association between benzene exposure and AML was observed in all our models suggests that the exposure assessment quality is sufficient, and the limitations do not preclude our study from finding other exposure–disease associations (25). Additionally, we could not account for lifestyle factors such as smoking and occupational exposures other than benzene. However, adjustment for factors such as sex, age, and education in our full model may partially mitigate the effect of potential confounders such as smoking. With notable exception of trichloroethylene (TCE), the evidence of other solvents being related to LH cancers is limited. Because correlation of TCE and benzene exposure is generally low, we do not expect confounding by TCE exposure in the observed associations. We acknowledge that our exposure metric for the continuous model differ from cumulative exposures calculated from complete occupational histories and may not be transferrable to other cohorts with different exposure metrics.

Our study also has several strengths. The Swiss National Cohort is a large cohort with information on nearly three million eligible participants collected in two mandatory censuses. Switzerland has a compulsory health insurance system with universal healthcare. The large cohort size allowed for analyses of 3055 benzene-exposed LH cancer cases. For occupational studies investigating risks of rare LH cancer subtypes, our study is one of the largest in terms of exposed cases, including >100 exposed cases each of DLBCL and HL, and >40 exposed cases each of ALL, FL, and SCBCL. In addition, participation rates in the Swiss national censuses and successful linkage rates to death records were very high. Thus, our study population represents a nearly complete coverage of the Swiss population with minimal selection bias. Occupational exposure assessment was made with the application of an established quantitative JEM that has been applied in other studies, where relationships were found between occupational benzene exposure and different health outcomes (11, 12). The time axis is a critical JEM feature for increasing the validity of exposure assessment, as patterns of industrial benzene use and exposure have changed in the last few decades, leading to lower exposure in most workplaces around the world (13, 29). Time trends in BEN-JEM were estimated based on expert judgment, incorporating knowledge about changes of benzene in stock feeds (eg, gasoline), implementation of regulations, and occupational control measures.

Our work highlights that registry-based occupation and disease information may be used to study occupational diseases in the general population in conjunction with a population-based JEM. Our results provide evidence that occupational exposure to benzene is associated with elevated mortality risks for AML, DLBCL, and possibly FL in the Swiss population. Additional work is needed to replicate and extend these findings in other settings, particularly in studies with different populations and complete occupational histories for more detailed exposure assessment.
